# Angiogenic and Microvascular Status Alterations after Endovascular Revascularization of Lower Limb Arteries among Patients with Diabetic Foot Syndrome: A Prospective 12-Month Follow-Up Study

**DOI:** 10.3390/jcm12175581

**Published:** 2023-08-27

**Authors:** Martyna Schönborn, Iwona Gregorczyk-Maga, Krzysztof Batko, Mikołaj Maga, Katarzyna Bogucka, Katarzyna Gawlik, Dorota Pawlica-Gosiewska, Paweł Maga

**Affiliations:** 1 Department of Angiology, Faculty of Medicine, Jagiellonian University Medical College, 31-008 Krakow, Poland; mikolaj.maga@gmail.com (M.M.); maga.pawel@gmail.com (P.M.); 2 Doctoral School of Medical and Health Sciences, Jagiellonian University, 31-007 Krakow, Poland; 3 Clinical Department of Angiology, University Hospital in Krakow, 30-688 Kraków, Poland; kat.bogucka1@gmail.com; 4 Faculty of Medicine, Institute of Dentistry, Jagiellonian University Medical College, 31-008 Kraków, Poland; iwona.gregorczyk-maga@uj.edu.pl; 5 Department of Research and Development, Medicine Economy Law Society (MELS) Foundation, 30-040 Krakow, Poland; batko.krzysztof@gmail.com; 6 Department of Clinical Biochemistry, Jagiellonian University Medical College, 31-008 Krakow, Poland; k.gawlik@uj.edu.pl (K.G.); dorota.pawlica@uj.edu.pl (D.P.-G.)

**Keywords:** angiogenesis, microcirculation, angioplasty, revascularization, diabetic foot syndrome

## Abstract

Peripheral arterial disease (PAD)-induced ischemia is an important component of diabetic foot syndrome (DFS). The results of revascularization of the lower extremity arteries (including percutaneous transluminal angioplasty [PTA]) do not always give satisfactory long-term results, which is due to many factors. The aim of the study was to investigate the alterations in selected circulating angiogenic factors and microcirculation parameters in 41 patients with DFS following PTA and analyze their relationships with clinical outcomes during 1-year follow-up. Our study revealed a general decrease in pro-angiogenic factor levels after PTA and their subsequent stabilization during subsequent observation. The results indicated a significant association between plasma circulating FGF-2 level and poor outcomes (including the incidence of restenosis/reocclusion of treated arteries) during 12 months of observation. The perioperative changes in FGF-2 showed a significant association with LDF alterations after PTA. Follow-up 1–3 months post-intervention showed a tendency towards elevated TcpO2, VEGF-A, and VEGF-R2 levels in patients free from adverse events. These results may provide a basis for further research on the potential use of selected circulating angiogenic factors for monitoring the treatment of patients with DFS following PTA.

## 1. Introduction

Peripheral arterial disease (PAD) is characterized by progressive narrowing and occlusion of arteries in peripheral regions, resulting in critical ischaemia and potentially serious complications such as amputation of limbs [1,2]. The 2019 European Society for Cardiology guidelines indicate that around one-third of hospitalized patients diagnosed with PAD also have diabetes mellitus (DM) [3]. The prevalence of PAD in the general population is estimated to be between 10% and 26%, while in patients with DM, it ranges from 20% to 28%. On the other hand, among individuals with diabetic foot ulcerations (DFUs), the prevalence of PAD reaches up to 50% [4]. PAD in diabetic patients often remains asymptomatic and progresses rapidly due to various metabolic abnormalities associated with DM [5]. Additionally, it tends to be more widespread, affecting distal limb arteries such as the tibial and peroneal arteries, in contrast to non-diabetic PAD [2]. PAD-induced ischemia is an important component of one of the major complications of diabetes, namely diabetic foot syndrome (DFS). Approximately 15% of individuals diagnosed with DM are affected by DFS, which is strongly associated with lower-limb amputations, disability, poor quality of life, and premature death. Among patients with DFUs, an estimated 14–24% will undergo lower extremity amputation, and the mortality rate within five years following amputation is reported to be as high as 50–59% [6,7].

DFS etiology results from several underlying causes, including impaired wound healing, ischemia from PAD, neuropathy, trauma, and high plantar pressures. The extended and disrupted healing mechanism in individuals with DFS is attributed to the inadequate coordination of blood vessel formation, disturbed balance in angiogenic and growth factors, abnormally prolonged inflammation, as well as an impaired immune response overall [8,9]. In diabetic wounds, inadequate angiogenic response leads to diminished blood vessel formation and capillary density, causing a substantial deficiency of immune cells, nutrients, and oxygen in regenerating tissues [7].

The therapy of DFS is multidirectional and relies on the collaboration of specialists from various fields who continuously seek new complementary forms of treatment that could influence the impaired wound healing process. As PAD implicates the development of tissue loss in up to half of patients with DFUs, revascularization (including percutaneous transluminal angioplasty [PTA]) should be considered early and sometimes among patients with large or infected wounds, regardless of the apparent perfusion deficit, because of the high risk of limb loss [10]. However, studies have demonstrated that diabetic patients had poorer outcomes in terms of perioperative complications, amputation rates, and mortality rates compared with non-diabetic ones [4]. Regarding ulcer healing during post-revascularization follow-up, studies indicated that approximately 60% of patients achieve healing within 1 year with either open bypass surgery or endovascular revascularization [11]. However, even up to 40% of patients who achieve wound healing can report a new or recurrent ulcer within 12 months [12]. The pathomechanism of this phenomenon is complex, multifactorial, and still not fully understood. Therefore, it seems justified to search for biomarkers that could serve as predictors of endpoints such as ulcer healing or amputation. Considering the significant role of angiogenesis in the process of proper wound healing, it appears reasonable to search for such markers among angiogenic factors. To the best of our knowledge, there is currently a lack of research regarding the changes in angiogenic factors after endovascular revascularization of lower limb arteries.

The aim of this study was to investigate the alterations in selected circulating pro- and anti-angiogenic factors and microcirculation parameters in patients with DFS following PTA of lower limb arteries. Additionally, we aimed to assess the association between these factors and the clinical outcomes, including ulcer healing as well as endpoints such as new ulcer formation, treated artery reocclusion/restenosis, limb amputation, the need for additional angioplasty, myocardial infarction, or all-cause death, during 12 months of follow-up observation.

## 2. Materials and Methods

### 2.1. Study Design and Population

We performed a single-centered, prospective cohort study including patients from the Clinical Department of Angiology between February 2021 and May 2022. The inclusion criteria were as follows: (1) subjects aged 40–80 years old; (2) diabetes type 2; (3) DFS with active ulcerations; (4) concomitant critical limb ischemia due to PAD (category 5 or 6 in the Rutherford classification); and (5) successful endovascular treatment of lower limb arteries. Patients diagnosed with Charcot’s foot, acute lower limb ischemia within the previous 3 months, myocardial infarction or stroke within the last 6 months, chronic kidney disease with eGFR < 45 mL/min/1.73 m^2^, neoplasm diagnosed within 5 years, or lacking the possibility of follow-up participation were excluded from participation. The study did not include subjects with chronic infectious diseases or autoimmune comorbidities.

All study participants underwent a 1-year observation period following PTA. Study visits were scheduled at 1, 3, 6, and 12 months after hospitalization. At each visit, the clinical status of the lower limbs and progression/regression of DFS syndrome were assessed, along with the evaluation of hemodynamic and microcirculatory parameters. Endpoints including restenosis or reocclusion of the treated artery, new ulcer formation, the need for additional PTA, major lower limb amputation, myocardial infarction, and all-cause death were meticulously recorded. A composite outcome of poor prognosis was determined based on the occurrence of at least one of the aforementioned unfavorable events.

### 2.2. Angiography, PTA of Lower Limb Arteries, and Post-Revascularization Pharmacological Therapy

All patients in the study group underwent PTA of the lower limb arteries at the Angiology Department Cath Lab. No general anesthesia or surgical incision was performed. Low-ionic and low-osmolality contrast agents were used during the PTA. The medical equipment and PTA procedure remained unchanged for the study. The decision to use this treatment, including the consideration of endovascular stent implantation, was independent of this research. The pharmacological therapy was carefully standardized, following current guidelines [13] and the center’s experience. After a balloon angioplasty, patients received a single antiplatelet drug, typically ASA 75 mg, along with subcutaneous low-molecular-weight heparin (LMWH) at a dose of 40 mg for one month. For those undergoing bare-metal stent (BMS) implantation, a dual antiplatelet therapy (DAPT) of ASA 75 mg and clopidogrel 75 mg, along with subcutaneous LMWH at a dose of 40 mg for one month, was administered. In some cases, a drug-eluting stent (DES) implantation required extended DAPT for 3 months. Eight patients with atrial fibrillation were on chronic anticoagulant therapy with dabigatran, either 110 mg or 150 mg twice daily. Importantly, this treatment approach had no impact on the study’s results.

### 2.3. DFS Advancement Evaluation

The assessment of the chronic limb ischemia category at baseline and during follow-up visits was conducted based on the symptomatic Rutherford classification [14]. To objectively assess and standardize the evaluation of DFS severity, two scales recommended by the International Working Group on the Diabetic Foot (IWGDF) were utilized: the WIfI and SINBAD classification systems [15]. The WIfI system involves the assessment of the wound’s presence, insensitivity to ischemia, and infection status using four severity grades for each category. It allows for the determination of 64 different clinical combinations and provides an estimated risk of amputation at 1 year [16]. The SINBAD classification grades six elements: ulcer site, ischemia, neuropathy, bacterial infection, area, and depth. The individual components of the SINBAD classification can be combined to generate a score ranging from 0 to 6 [17].

### 2.4. Hemodynamic Parameters and Microcirculation Assessment

Lower limb ischemia was assessed using the ankle-brachial index (ABI) and toe-brachial index (TBI). A sphygmomanometer, an 8 MHz blind Doppler flow detector, and digital plethysmography were used to measure systolic blood pressure in the brachial arteries, ankle arteries, and toe capillaries. Measurements were conducted in controlled conditions at a room temperature of 21–23 °C, following a 15-min rest in a supine position with parallel limbs [18].

Microcirculation was evaluated using a transcutaneous oxygen pressure test (tcpO2) and laser Doppler flowmetry (LDF). The Periflux 6000 device (Perimed AB, Järfälla, Sweden) was employed for both assessments, equipped with thermostatic laser Doppler probes for precise tissue heating at the measurement site and a modified Clark’s polarographic oxygen sensor. Probes were positioned on the distal foot’s dorsal aspect, excluding areas with inflammation, necrotic tissue, bone prominence, or superficial tendons. Baseline microvascular blood flow was recorded for 5 min and expressed in arbitrary perfusion units (PU). The tcpO2 test lasted for 20 min or until a flattened curve appeared on the graph, with results presented in mmHg. These findings encompassed microvascular flow, including capillaries, arterioles, venules, and shunts, as well as microcirculatory blood perfusion, metabolic activity, oxyhemoglobin dissociation, and tissue oxygen partial pressure. Both examinations were conducted in a room with a temperature of 21–23 °C, following a 15-min rest in a supine, comfortable position for the patient.

### 2.5. Circulating Angiogenic Factors Assessment

Angiogenic processes were evaluated by analyzing a selected set of seven naturally occurring circulating biomarkers with either pro- or anti-angiogenic properties. Extra blood samples were collected from all subjects 1 day before PTA, 1 day after PTA (pre- and post-intervention changes), as well as during follow-up observation—1 and 3 months after the intervention. The set of five pro-angiogenic biomarkers included vascular endothelial growth factor A (VEGF-A), soluble vascular endothelial growth factor receptor 2 (VEGF-R2 or sVEGF-R2), fibroblast growth factor 2 (FGF-2 or basic fibroblast growth factor), placental growth factor (PlGF), and platelet-derived growth factor-BB (PDGF-BB). Additionally, the evaluation encompassed two anti-angiogenic factors: pigment epithelium-derived factor (PEDF) and angiopoietin-1 (ANG-1). The main material for angiogenic factor assessment was plasma separated from ethylenediaminetetraacetic (EDTA) whole-fasting blood samples after centrifugation at 3000 rpm for 15 min. All samples were stored at −80 °C until analysis. Human VEGF-A and VEGF-R2 concentrations (pg/mL) were determined using an enzyme-linked immunosorbent assay with commercially available ELISA kits (Thermo Fisher Scientific, Inc., Waltham, MA, USA; Cat. No. BMS277 and BMS2019). Human PDGF-BB, ANG-1, FGF, and PIGF levels (pg/mL) were measured via the quantitative sandwich enzyme immu-noassay technique (R&D Systems, Minneapolis, MN, USA; Cat. No. DBB00, DANG10, DFB50, and DPG00). Human PEDF concentration (µg/mL, but data shown in ng/mL) was determined via an immunoenzymatic method using a commercially available ELISA kit (BioVendor—Laboratorni Medicina a.s., Brno, Czech Republic, Cat. No. RD191114200R).

Additional samples of unstimulated whole saliva (WS, collected after a fasting period) were obtained to evaluate the concentrations of VEGF-A, PDGF-BB, and ANG-1 and compare their levels in saliva with those in plasma at three different time points (at baseline, 24 h and 1 month after the PTA). The measurements were conducted using the same enzyme-linked immunosorbent assays employed for plasma analysis. The samples were collected in 10 mL plastic tubes. All materials were stored at −80 °C until the final analysis.

All relevant variables that could potentially influence neovascularization and affect both early and long-term outcomes were carefully considered. Biomarkers associated with established measures of glycemic and lipid control, inflammation, and renal function were examined, including hemoglobin level, platelet count (PLT), white blood cell count (WBC), creatinine, C-reactive protein (CRP), glycated hemoglobin (HbA1c), total cholesterol (TC), triglycerides (TG), high-density lipoprotein cholesterol (HDL-C), and low-density lipoprotein cholesterol (LDL-C).

### 2.6. Statistical Analysis

Analysis was performed in R 4.2.2 (R Core Team, 2023, Vienna, Austria) using publicly available packages. Data were summarized using the mean (standard deviation; SD) or median (interquartile range; IQR). The variable distribution was assessed using density plots with visual inspection. Comparison was performed using a *t*-test or Kruskal–Wallis test, as deemed appropriate. Linear relationships were assessed using Pearson’s correlation and are provided in reference to scatter plots with linear fit. The visualization was performed with ggplot. Due to the constraints of the sample size, most analyses are univariable, with limited capacity for inter-group breakdown. Tests were two-tailed, and a *p* value < 0.05 was considered statistically significant. In order to account for age as a confounding variable in the measurement results, the basic characteristics of the group and the concentrations of angiogenic biomarkers were also analyzed by dividing the participants into two age groups (≤65 years and >65 years).

## 3. Results

### 3.1. Demographic and Clinical Characteristics of Subjects

The mean age of this cohort was 67.27 (8.84) years, with a male predominance in gender distribution (34, 82.9%). Hypertension (*n* = 37, 90.2%) was the most common comorbidity. Overall, fifteen (36.6%) subjects had coronary artery disease, and seven (17.1%) had a prior myocardial infarction. Nineteen subjects (46.3%) had previously experienced lower limb amputation (major or minor). A comparison of demographic and clinical characteristics by age group is provided in Table 1.

All patients had some form of lower limb ulceration at baseline. These ulcers were most often localized within the forefoot region (65%). At baseline, twenty-one (52.5%) cases were described as having a penetrating character, and twenty-three (57.5%) had concomitant signs of wound infection. For twenty-six (65%) individuals, the wound was described as deep or consisting of gangrenous tissue. Additional measures related to ulcer characteristics were obtained using SINBAD (median 5 [IQR 3–5]) and WiFi (median 6 [IQR 4–7]) scores. High-grade ischemia was observed in thirty subjects (93.8%). A mild to moderate grade of infection was reported among thirteen (31.7%) and 17 individuals (41.5%), respectively. High risk of amputation, according to WiFi classification, was observed for twenty-seven individuals (65.9%), while the rest was categorized as low to moderate risk (34.1%).

### 3.2. Changes in Circulating Angiogenic Factor Concentrations, Hemodynamic, and Microcirculatory Parameters over Follow-Up

In most cases, the mean levels of pro-angiogenic factors decreased immediately after the procedure and then remained at the same level during subsequent observation. All angiogenic factor levels in plasma at each timepoint are summarized using different measures of central tendency and distribution in Table 2.

Regarding the perioperative changes in hemodynamic and microcirculatory parameters, there was an increase immediately after the procedure, with further elevation in TcpO2 during both short-term follow-up (1 and 3 months) and long-term observation (6 and 12 months). However, for TBI and LDF, after an initial post-procedure increase, their values remained relatively stable during subsequent observation. LDF values were the highest immediately after the procedure. The change in ABI values was minimal, and the mean parameter values remained consistent throughout the entire observation period. All changes are summarized by timepoints and presented in Table 3.

### 3.3. Relationships between Baseline Plasma Angiogenic Factor Levels before PTA and Wound Healing during 12 Months According to Patients’ Age

During 12 months of follow-up, complete wound healing was achieved by 22 subjects (53.7 %). We further analyzed the relationships between baseline plasma angiogenic factor levels and wound healing, stratified by age group. Mean plasma FGF-2 was not associated with ulcer healing by 12 months (*p* = 0.786) or the development of new ulceration (*p* = 0.779). The mean plasma FGF-2 level at baseline was significantly higher among younger patients (≤65 years old) who experienced ulcer healing (*p* = 0.019) (Figure S1), but it was not observed among older subjects (>65 years old). No significant differences by age group or ulcer status were observed for the remaining angiogenesis-related factors (VEGF-A, VEGF-R2, PlGF, PDGF-BB, PEDF, and Ang-1).

Regarding the influence of glycemic control on ulcer healing, the mean HbA1c values were comparable between individuals with healed and non-healed ulcers after 12 months. Specifically, at baseline, the mean HbA1c was 7.86 (1.41%) for those with healed ulcers and 7.95 (1.85%) for those with non-healed ulcers after 12 months (*p* = 0.85). After 12 months, the mean HbA1c was 7.86 (1.73%) among patients with healed ulcers and 8.08 (1.71%) among those with non-healed ulcers (*p* = 0.70).

### 3.4. Relationships between Plasma Angiogenic Factors Levels before and after PTA and Poor Outcome after 12 Months

The composite poor outcome, consisting of new ulcer formation, lower limb amputation (major or minor), treated artery reocclusion/restenosis, the need for additional angioplasty, myocardial infarction, or all-cause death, was assessed at 12 months. Overall, nineteen patients (46.3%) met this primary endpoint. Of these, seven subjects (17.1%) experienced limb amputation (major or minor), and two patients died.

**Figure 1:**
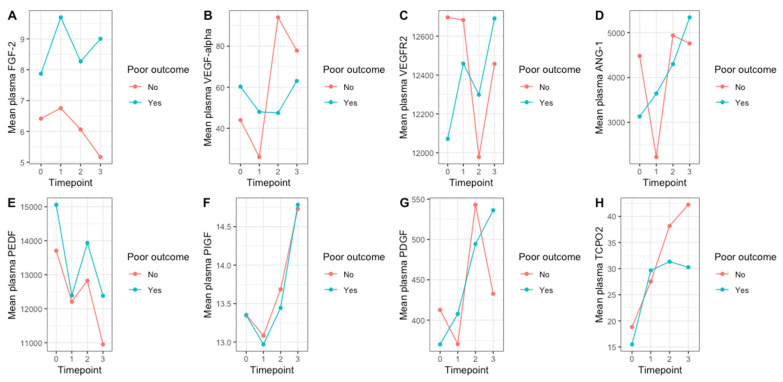
Line plots illustrating temporal changes in mean plasma angiogenic factor concentrations (**A**—FGF-2, **B**—VEGF-A, **C**—VEGF-R2, **D**—ANG-1, **E**—PEDF, **F**—PlGF, **G**—PDGF) and tcpO2 (**H**) at baseline and in three subsequent timepoints after PTA compared according to outcome status. A composite outcome of poor prognosis was determined based on the occurrence of at least one of the following: new ulcer formation, treated artery reocclusion/restenosis, limb amputation, the need for additional angioplasty, myocardial infarction, or all-cause death. VEGF-alpha—vascular endothelial growth factor alpha/A, VEGF-R2—vascular endothelial growth factor receptor 2, FGF-2—fibroblast growth factor 2, PlGF—placental growth factor, PDGF-BB—platelet-derived growth factor-BB, PEDF—pigment epithelium-derived factor, ANG-1—angiopoietin-1, tcpO2—transcutaneous oxygen pressure, timepoint 0—baseline, timepoint 1—24 h after PTA, timepoint 2—1 month after PTA, timepoint 3—3 months after PTA, poor outcome—new ulcer formation, treated artery reocclusion/restenosis, limb amputation, the need for additional angioplasty, myocardial infarction, or death.

We examined the temporal relationship between changes in plasma angiogenic factor levels at each timepoint and endpoint status (a visual representation is provided in Figure 1). At baseline and over follow-up, mean plasma FGF-2 concentrations were higher among subjects who experienced unfavorable outcomes. Furthermore, a trend towards higher values of TcpO2, VEGF-A, and VEGF-R2 was observed during long-term follow-up (1 month and 3 months post-intervention) among patients who remained free from adverse events. Regarding anti-angiogenic biomarkers, the average values of PEDF were significantly higher among patients with adverse outcomes throughout the entire observation period.

Regarding the impact of glycemic control on the composite poor outcome, the mean HbA1c values were comparable between individuals with poor outcomes and those without adverse events during the past 12 months. At baseline, the mean HbA1c was 8.07 (1.80%) for subjects who experienced unfavorable events and 7.75 (1.45%) for those without poor outcomes (*p* = 0.55). Similarly, after 12 months, the mean HbA1c was 8.31 (1.96%) among individuals with adverse events and 7.68 (1.44%) among those without unfavorable events (*p* = 0.29).

### 3.5. Relationships between Mean Plasma FGF-2 Levels, Restenosis/Reocclusion of Treated Artery, and Ulcer Healing 12 Months after PTA

**Figure 2:**
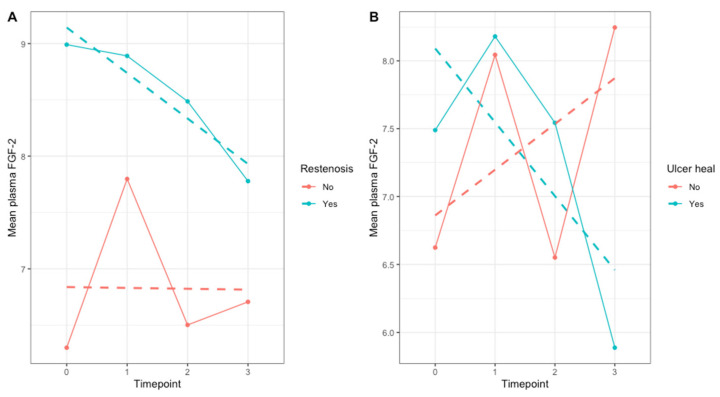
Lineplot illustrating changes in mean plasma FGF-2 stratified by restenosis/reocclusion (**A**) or ulcer status (**B**). Fixed points reflect mean values at a given timepoint, while dashed lines reflect a linear fit using a univariable model for time. Endpoint status is defined as occurrence of a given event after PTA according to restenosis/reocclusion of treated artery or ulcer healing during 12 months after PTA. Mean plasma marker values reflect the average over three timepoints. FGF-2—fibroblast growth factor 2, timepoint 0—baseline, timepoint 1—24 h after PTA, timepoint 2—1 month after PTA, timepoint 3—3 months after PTA.

The prognostic value of plasma FGF-2 concentrations was further explored due to their relationship with the occurrence of poor outcomes. During 12 months of follow-up, treated artery restenosis/reocclusion was experienced by twelve patients (29.3%). FGF-2 levels shared a particularly visible relationship with the occurrence of artery restenosis or reocclusion, which was a constituent element of the composite poor outcome (Figure 2A). Higher FGF-2 values at each time point tended to differentiate patients who experienced restenosis/reocclusion compared with patients without this adverse event. In contrast, greater variability in mean plasma FGF-2 levels and ulcer healing by 12 months is observed (Figure 2B). While reliance on linear fit would suggest a decline in FGF-2 values is tied to wound improvement, the variability between timepoints likely precludes practical utility. Mean plasma FGF-2 values were higher among patients who achieved ulcer healing only up to the first month, which can suggest confounding of this relationship by other factors given a longer period of observation. Age may be an additional confounder of this association (see Figure S1).

### 3.6. Relationships between Pre- and Post-Intervention Changes in LDF and Angiogenic Factor Concentrations

We investigated the relevance of change rates (defined as a proportion of post-intervention values to pre-intervention values) in parameters that reflect microcirculatory impairment, wound ischemia, and angiogenesis. In univariable analyses, a significant association for changes in LDF (R = 0.35, *p* = 0.037) was observed for delta FGF-2 (see Figure S2).

### 3.7. Relationship between VEGF-A, PDGF-BB, and ANG-1 Levels in WS and Plasma at Three Time Points

We also studied salivary concentrations of the three plasma angiogenic biomarkers (VEGF-A, PDGF-BB, and ANG-1) at three time points—at baseline, 24 h after PTA, and 1 month after PTA—but there was no consistency between assay values.

## 4. Discussion

In our study, we focused on the dynamics of changes in concentrations of pro- and anti-angiogenic factors after PTA and their correlations with both ulcer healing and adverse endpoints during a one-year follow-up. To our knowledge, no studies have been published analyzing changes in such a wide range of angiogenic biomarkers following peripheral arterial revascularization procedures. Our results demonstrated a tendency for a decrease in the concentrations of the mentioned pro-angiogenic biomarkers in the short-term period after the procedure (24 h), followed by their stabilization during subsequent observation. Unfortunately, it is challenging to compare these findings with those of other authors due to the limited number of available studies on this topic. Only one study showed that surgical revascularization in patients with symptomatic PAD did not alter circulating VEGF 10 days after surgery [19]. More extensive literature has been conducted on the topic of angiogenic factors in coronary artery disease (CAD), resulting in a substantial body of literature available. The study investigating changes in VEGF and vascular endothelial growth factor receptor 1 (VEGF-R1) levels 6 days after myocardial revascularization revealed an increase in VEGF levels from 192.4 ± 166.1 pg/mL to 264.7 ± 226.6 pg/mL following the intervention [20]. Moreover, circulating levels of total VEGF-A were shown to be correlated with CAD complexity, while VEGF-A was significantly higher among patients with higher syntax scores (SS) compared with the low SS group [21].

Based on the experiences of our department and the identified association between the salivary and blood levels of the PEDF factor in patients with PAD [22], we also decided to measure selected biomarkers in both WS and plasma in our study. However, the results demonstrated no consistency between assay values. Several factors could have influenced the result. Saliva, despite several advantages as a diagnostic material [23], is not always easy to collect, especially in older patients with comorbidities. This may be attributed to the commonly reported dry mouth (xerostomia), which is also prevalent in patients with diabetes [24]. This issue, along with poor oral hygiene, could have potentially affected the results obtained.

In our study population, during the one-year follow-up, restenosis or reocclusion occurred in almost one-third of patients, and in all cases, it necessitated further endovascular intervention. This value is slightly lower than that reported in previous studies [25,26], which may be attributed to the small size of the analyzed patient group. Restenosis, defined as the recurrence of significant arterial lumen narrowing following revascularization, occurs due to endothelial damage, which leads to the proliferation of neointimal cells and vascular smooth muscle cells (VSMCs) [27]. Diabetic patients are at increased risk of restenosis, and neointimal hyperplasia looks phenotypically different from the one observed in non-diabetic subjects. It results from greater adhesion and migration of VSMCs with increased cell mitogenesis stimulated by pro-inflammatory cytokines [28,29]. This knowledge is consistent with accelerated rates of coronary narrowing and thrombosis and the need for repeat revascularization in diabetic patients [30,31].

The main finding from our analysis is the clear association between circulating FGF-2 levels and adverse events in patients with DFS who underwent endovascular revascularization of lower limb arteries. Specifically, we observed a direct relationship between mean FGF-2 concentrations and the composite poor endpoint, which also included the occurrence of restenosis or reocclusion in treated arteries. These results are consistent with our previous study, where we demonstrated a significant correlation between circulating FGF-2 levels and more advanced DFS [32]. Increasingly, there are indications in the literature regarding the dual nature of FGF-2, depending on whether the biomarker is present in circulating form or in tissues. FGF-2, despite its obvious proangiogenic properties [33], also plays a role in atherosclerotic lesion growth and inflammatory processes. It can induce intimal thickening, intraplaque angiogenesis, and VSMC proliferation [34,35]. This phenomenon has become the basis for studies on the relationship between fibroblast growth factors (FGFs) and CAD [36,37]. It was shown that patients with ischemic heart disease exhibit elevated serum levels of bFGF [38]. Moreover, it has been revealed that elevated plasma levels of FGF-2 could potentially serve as a predictive biomarker for the development of CAD in adult males with DM type 2 [39].

In addition, our analysis indicated a noticeable trend towards higher values of TcpO2, VEGF-A, and VEGF-R2 during mid-term follow-up (1 month and 3 months post-intervention), as well as lower concentrations of PEDF during the entire observation, among patients who remained free from poor adverse events. In the literature, a positive association between baseline TcpO2 values and the degree of ulcer healing with intact skin was revealed [40]. Furthermore, one previous study demonstrated that TcpO2 is a more reliable predictor of preoperative limb ischemia and postoperative outcomes of revascularization compared with hemodynamic assessments by Doppler-derived pressure tests [41].

According to ulcer healing during the observation, it was achieved by 22 subjects (53.7%). This finding is in line with the updated systematic review by the International Working Group on the Diabetic Foot (IWGDF) on the efficacy of revascularization in ulcerated feet. It demonstrated that wound healing was achieved in 60% of patients (with an interquartile range of 50–69%) at the one-year follow-up when treated with either endovascular or surgical interventions [42]. Regarding the correlation between angiogenic factors and healed ulcers, the results of our study revealed challenges in their interpretation. It was found that the relationships between circulating angiogenic biomarker levels and healed ulcerations are significantly influenced by the age of patients. Only one significant correlation for the mean plasma FGF-2 level at baseline was found, but it was not observed among the elderly participants.

The healing of ulcers among diabetic patients is influenced by far more factors than only changes in angiogenesis processes or alterations in microcirculation function. In the context of glycemic control and its impact on ulcer healing, our findings indicated that HbA1c values did not exhibit a significant difference between the two groups in relation to ulcer healing after the 12-month period. An important issue to address is the impact of chronic kidney disease (CKD), one of the complications of diabetes, on the wound healing process. The study conducted by Caruso et al. demonstrated that among patients with non-healed ulcers during a 12-month follow-up, there was a notable increase in urinary albumin excretion levels and a significant rise in the prevalence of diabetic CKD [43]. In our study, we excluded patients with eGFR < 45 mL/min/1.73 m^2^ to ensure a more homogenous subject group and to avoid the potential influence of kidney impairment on ulcer healing. Another factor that undoubtedly affects ulcer healing is the presence of infection in the healing tissues. Among patients with DFS, antibiotic therapy and the associated issue of antibiotic resistance should always be considered during multidisciplinary care. This is supported by the study by Caruso et al., which demonstrated that antibiotic resistance occurs in up to 36% of cases of diabetic foot infection (DFI), and during the COVID-19 pandemic, these rates significantly increased to as high as 63% [44]. In our study, we had inadequate data to analyze this matter. The absence of comprehensive antibiograms for ulcer infection cases is due to the local therapy being administered at multiple centers, often in remote regions of the country.

The study has its limitations. First of all, the study population is relatively small, which could impact the results, especially in the aspect of adverse vascular event analysis. There is also no control group, including participants with PAD but without diabetes, which determines that the obtained results and conclusions can be applied only to patients with diabetic foot syndrome and concomitant PAD.

## 5. Conclusions

Our study revealed a general decrease in pro-angiogenic factor concentrations after PTA and their subsequent stabilization during subsequent observation. The results indicated a significant correlation between plasma circulating FGF-2 levels and poor outcomes during 12 months of observation. Baseline and follow-up analyses showed higher plasma FGF-2 concentrations in subjects with restenosis/reocclusion of treated arteries. Moreover, 1–3 months of follow-up post-intervention showed a tendency towards elevated TcpO2, VEGF-A, and VEGF-R2 levels in patients free from adverse events. These results emphasize the strong relationship between angiogenesis and the process of wound healing in patients suffering from DFS. It may provide a basis for further research on the potential use of selected circulating angiogenic factors for monitoring the treatment of patients with DFS following PTA.

## Figures and Tables

**Table 1:** Baseline characteristics, concomitant disorders, hemodynamic parameters, and microvascular status according to patients’ age.

Variable	≤65 Years (*n* = 15)	>65 Years (*n* = 26)	Total (*n* = 41)	*p* Value
Age, years				<0.01
mean (SD)	57.67 (4.73)	72.81 (5.03)	67.27 (8.84)	
median (IQR)	58.00 (55.00, 61.50)	72.50 (69.00, 74.75)	68.00 (61.00, 73.00)	
sex				
male	13 (86.7%)	21 (80.8%)	34 (82.9%)	1.00
BMI, kg/m^2^				0.19
mean (SD)	28.48 (3.74)	26.98 (3.24)	27.55 (3.47)	
median (IQR)	28.73 (26.01, 31.39)	26.01 (24.70, 29.48)	26.87 (24.70, 29.48)	
active or past smoker	12 (80.0%)	17 (65.4%)	29 (70.7%)	0.48
Concomitant disorders				
arterial hypertension	13 (86.7%)	24 (92.3%)	37 (90.2%)	0.61
CAD	5 (33.3%)	10 (38.5%)	15 (36.6%)	1.00
previous MI	4 (26.7%)	3 (11.5%)	7 (17.1%)	0.39
atrial fibrillation	2 (13.3%)	6 (23.1%)	8 (19.5%)	0.69
CKD	1 (6.7%)	3 (11.5%)	4 (9.8%)	1.00
eGFR, mL/min/1.73 m^2^				0.20
mean (SD)	84.14 (16.09)	76.00 (20.15)	78.85 (19.03)	
median (IQR)	90 (86.25, 90.00)	77.00 (61.50, 89.75)	84.50 (65.25, 90.00)	
Used medications				
insulin	12 (80.0%)	21 (80.8%)	33 (80.5%)	1.00
metformin	10 (66.7%)	18 (69.2%)	28 (68.3%)	1.00
SGLT-2 inhibitor	5 (33.3%)	5 (19.2%)	10 (24.2%)	0.45
GLP-1 agonist	0 (0.00%)	2 (7.7%)	2 (4.9%)	0.52
acarbose	0 (0.00%)	1 (3.8%)	1 (2.4%)	1.00
statins	7 (46.7%)	17 (65.4%)	24 (58.5%)	0.33
ASA/clopidogrel/both	8 (53.3%)	16 (61.5%)	24 (58.5%)	0.74
Hemodynamic parameters and microvascular status				
Baseline ABI				0.26
mean (SD)	0.74 (0.49)	1.01 (0.81)	0.91 (0.71)	
median (IQR)	0.67 (0.49, 0.87)	0.73 (0.46, 1.00)	0.72 (0.46, 1.00)	
Baseline TBI				0.72
mean (SD)	0.18 (0.15)	0.16 (0.09)	0.17 (0.12)	
median (IQR)	0.16 (0.06, 0.23)	0.14 (0.11, 0.20)	0.15 (0.11, 0.21)	
Baseline LDF, PU				0.11
mean (SD)	11.57 (3.55)	13.92 (4.95)	13.06 (4.59)	
median (IQR)	11.00 (8.75, 14.00)	13.75 (10.12, 16.75)	12.00 (9.00, 16.00)	
Baseline tcpO2, mmHg				0.49
mean (SD)	15.50 (9.97)	18.31 (13.23)	17.32 (12.13)	
median (IQR)	12.50 (9.00, 24.50)	15.00 (10.00, 26.25)	14.50 (9.75, 25.25)	

BMI—body mass index, eGFR—estimated glomerular filtration rate, CAD—coronary artery disease, MI—myocardial infarction, CKD—chronic kidney disease, ASA—acetylsalicylic acid, SGLT-2—sodium-glucose co-transporter-2, GLP-1—glucagon-like peptide 1, ABI—ankle-brachial index, TBI—toe-brachial index, tcpO2—transcutaneous oxygen pressure, LDF—laser doppler flowmetry, PU—perfusion units, IQR—interquartile range, SD—standard deviation.

**Table 2:** Angiogenic factor concentrations in plasma before PTA and at 3 time points after PTA (24 h, 1 month, and 3 months after PTA).

Factor	Before PTA (Timepoint 0)	24 h after PTA (Timepoint 1)	1 Month after PTA (Timepoint 2)	3 Months after PTA (Timepoint 3)
FGF-2, pg/mL				
mean (SD)	7.09 (5.06)	8.12 (6.72)	7.08 (6.74)	7.04 (6.86)
median (IQR)	6.07 (3.52–9.42)	5.76 (3.52–10.6)	4.17 (3.19–8.32)	4.49 (3.19–7.50)
range (min–max)	0.53–20.3	0.53–27.1	0.53–30.1	0.53–30.6
VEGF-A, pg/mL				
mean (SD)	51.5 (75.8)	36.2 (63.0)	72.5 (116.0)	70.6 (118.0)
median (IQR)	19.1 (3.67–55.6)	16.2 (2.84–35.7)	30.7 (3.67–86.7)	15.2 (3.67–90.8)
range (min–max)	0.51–298.0	0.51–357.0	1.01–587.0	0.51–557.0
VEGF-R2, pg/mL				
mean (SD)	12,407.0 (2723.0)	12,579.0 (2784.0)	12,127.0 (2626.0)	12,571.0 (3288.0)
median (IQR)	11,928.0 (10,540–13,801.0)	12035.0 (10,647.0–14,458.0)	11,819.0 (10,391.0–13,204.0)	12,292.0 (9951.0–14,667.0)
range (min–max)	7308.0–19,119.0	8078.0–18,557.0	8067.0–19,298.0	7308.0–19,575.0
PlGF, pg/mL				
mean (SD)	13.4 (3.31)	13.0 (3.46)	13.6 (3.71)	14.8 (5.78)
median (IQR)	12.3 (10.8–15.5)	12.5 (10.7–15.2)	13.7 (10.4–15.8)	13.4 (11.6–16.4)
range (min–max)	7.74–24.5	6.55–21.0	7.17–22.1	6.02–38.4
PDGF-BB, pg/mL				
mean (SD)	393.0 (357.0)	388.0 (334.0)	521.0 (464.0)	483.0 (463.0)
median (IQR)	300.0 (215.0–472.0)	249.0 (165.0–534.0)	377.0 (240.0–627.0)	353.0 (181.0–506.0)
range (min–max)	22.4–1887.0	40.3–1492.0	84.5–2093.0	51.1–2100.0
PEDF, µg/mL				
mean (SD)	14,334.0 (6566.0)	12,292.0 (5258.0)	13,340.0 (6703.0)	11,647.0 (4644.0)
median (IQR)	13,870.0 (9721.0–17,803.0)	11,773 (9307.0–15,083.0)	11,962.0 (8066.0–18,361.0)	10,720.0 (7497.0–14,818.0)
range (min–max)	5274.0–41,798.0	345.0–28,376.0	368.0–28,328.0	5087.0–23,276.0
Ang-1, pg/mL				
mean (SD)	3855.0 (4611.0)	2882.0 (2949.0)	4641.0 (3642.0)	5041.0 (4257.0)
median (IQR)	2744.0 (1662.0–4033.0)	1855.0 (1223.0–3468.0)	3955.0 (19.21–6970.0)	4039.0 (15.60–7090.0)
range (min–max)	296.0–28,908.0	316.0–16,210.0	719.0–18,907.0	475.0–18,134.0

VEGF-A—vascular endothelial growth factor A, VEGF-R2—vascular endothelial growth factor receptor 2, FGF-2—fibroblast growth factor 2, PlGF—placental growth factor, PDGF-BB—platelet-derived growth factor-BB, PEDF—pigment epithelium-derived factor, Ang-1—angiopoietin-1, IQR—interquartile range, SD—standard deviation.

**Table 3:** Microcirculatory parameter values before PTA and at 3 time points after PTA (24 h, 1 month, and 3 months after PTA).

Parameter	Before PTA	24 h	1 M	3 M	6 M	12 M
tcpO2, mmHg						
mean (SD)	17.3 (12.1)	28.5 (17.7)	35.1 (16.4)	36.7 (18.0)	37.3 (15.7)	44.9 (14.8)
median (IQR)	14.5 (9.75–25.2)	27.5 (15.0–37.8)	40.0 (26.8–44.2)	40.0 (23.0–48.0)	40.0 (22.0–50.0)	46.0 (34.0–55.0)
range (min–max)	0.0–51.0	0.0–80.0	2.0–62.0	3.0–78.0	7.0–62.0	6.0–70.0
LDF, PU						
mean (SD)	13.1 (4.59)	19.7 (15.5)	18.3 (10.2)	15.9 (7.09)	15.1 (6.01)	17.2 (6.95)
median (IQR)	12.0 (9.0–16.0)	16.0 (10.8–22.8)	15.5 (11.8–24.0)	14 (11.0–20.0)	13.5 (11.0–17.5)	16.0 (13.2–20.0)
range (min–max)	7.0–26.0	6.0–84.0	6.0–61.0	7.0–38.0	8.0–29.0	7.0–35.0
ABI						
mean (SD)	0.91 (0.71)	1.08 (0.52)	1.05 (0.51)	1.15 (0.74)	1.16 (0.88)	0.99 (0.68)
median (IQR)	0.73 (0.46–1.0)	0.92 (0.82–1.2)	0.95 (0.81–1.07)	0.89 (0.81–1.05)	0.83 (0.66–1.06)	0.83 (0.68–0.95)
range (min–max)	0.13–3.0	0.36–3.0	0.56–3.0	0.44–3.0	0.28–3.0	0.28–3.0
TBI						
mean (SD)	0.17 (0.12)	0.33 (0.18)	0.38 (0.24)	0.34 (0.25)	0.35 (0.25)	0.39 (0.21)
median (IQR)	0.16 (0.11–0.21)	0.28 (0.19–0.42)	0.35 (0.21–0.57)	0.32 (0.19–0.53)	0.37 (0.16–0.48)	0.39 (0.25–0.5)
range (min–max)	0.0–0.57	0.11–0.73	0.0–0.93	0.0–0.8	0.0–0.94	0.0–0.92

ABI—ankle-brachial index, TBI—toe-brachial index, tcpO2—transcutaneous oxygen pressure, LDF—laser doppler flowmetry, PU—perfusion units, IQR—interquartile range, SD—standard deviation.

## Data Availability

Data sharing is not applicable to this article.

## Supplementary Materials

The following supporting information can be downloaded at: https://www.mdpi.com/article/10.3390/jcm12175581/s1. Figure S1. Boxplot comparison of baseline angiogenic factors levels (A—FGF-2, B—VEGF-A, C—VEGF-R2, D—ANG-1, E—PEDF, F—PlGF, G—PDGF), baseline tcpO2 (H) and ulceration status 12 months after PTA according to patient ages. VEGF-alpha—vascular endothelial growth factor alpha/A, VEGF-R2—vascular endothelial growth factor receptor 2, FGF-2—fibroblast growth factor 2, PlGF—placental growth factor, PDGF—platelet-derived growth factor-BB, PEDF—pigment epithelium-derived factor, ANG-1—angiopoietin-1, tcpO2—transcutaneous oxygen pressure. Figure S2. Scatter plots with linear fit illustrating the relationship between changes in pre- to post-intervention changes in LDF and tcpO2 (A) and angiogenic factor concentrations (BPEDF, C—PlGF, D—FGF-2, E—ANG-1, F—VEGF-A, G—VEGF-R2, H—PDGF-BB). VEGF-A—vascular endothelial growth factor alpha/A, VEGF-R2—vascular endothelial growth factor receptor 2, FGF-2—fibroblast growth factor 2, PlGF—placental growth factor, PDGF-BB—platelet-derived growth factor-BB, PEDF—pigment epithelium-derived factor, ANG-1—angiopoietin-1, tcpO2—transcutaneous oxygen pressure, LDF—laser doppler flowmetry.
