# Supplementary material for: Angiogenic and Microvascular Status Alterations after Endovascular Revascularization of Lower Limb Arteries among Patients with Diabetic Foot Syndrome: A Prospective 12-Month Follow-Up Study
Source: J Clin Med. 2023 Aug 27;12(17):5581. doi: 10.3390/jcm12175581 (PMC10488381; doi:10.3390/jcm12175581)
Supplement: Supplementary file 1 [file jcm-12-05581-s001.zip › jcm-2534218-supplementary.pdf]

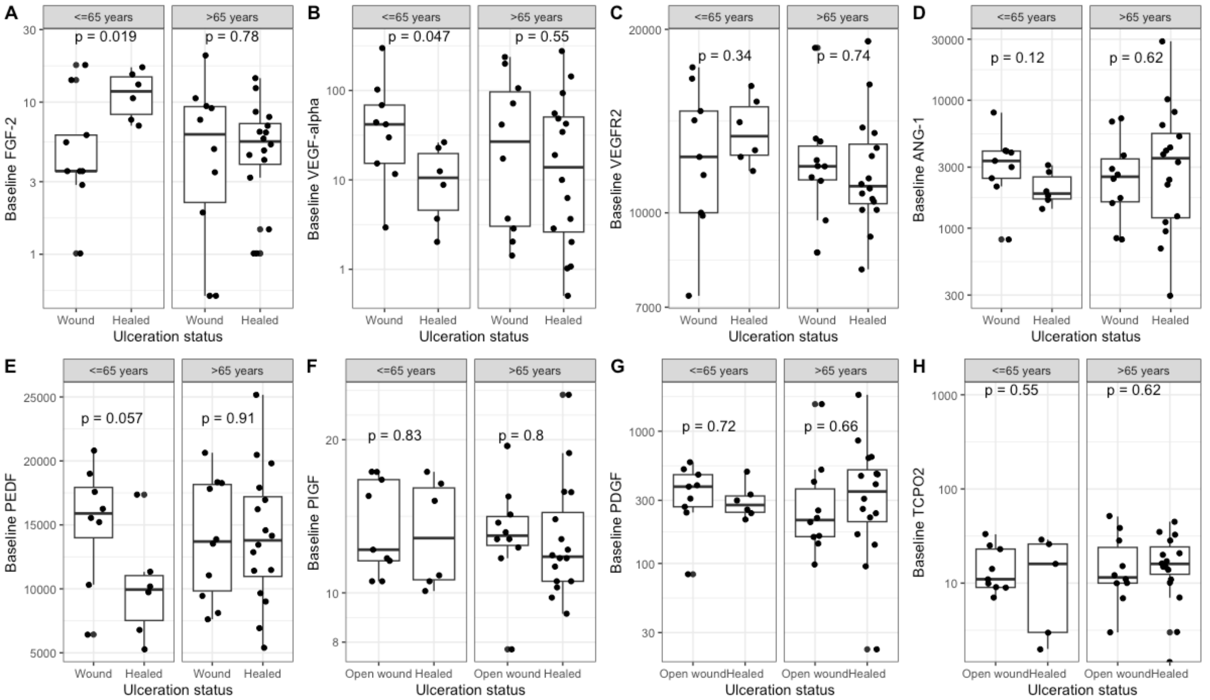

Figure S1. Boxplot comparison of baseline angiogenic factors levels (**A** – FGF-2, **B** – VEGF-A, **C** – VEGF-R2, **D** - ANG-1, **E** – PEDF, **F** – PlGF, **G** – PDGF), baseline tcpO2 (**H**) and ulceration status 12 months after PTA according to patient ages. VEGF-alpha—vascular endothelial growth factor alpha/A, VEGF-R2—vascular endothelial growth factor receptor 2, FGF-2—fibroblast growth factor 2, PlGF—placental growth factor, PDGF—platelet-derived growth factor-BB, PEDF—pigment epithelium-derived factor, ANG-1—angiopoietin-1, tcpO2—transcutaneous oxygen pressure.

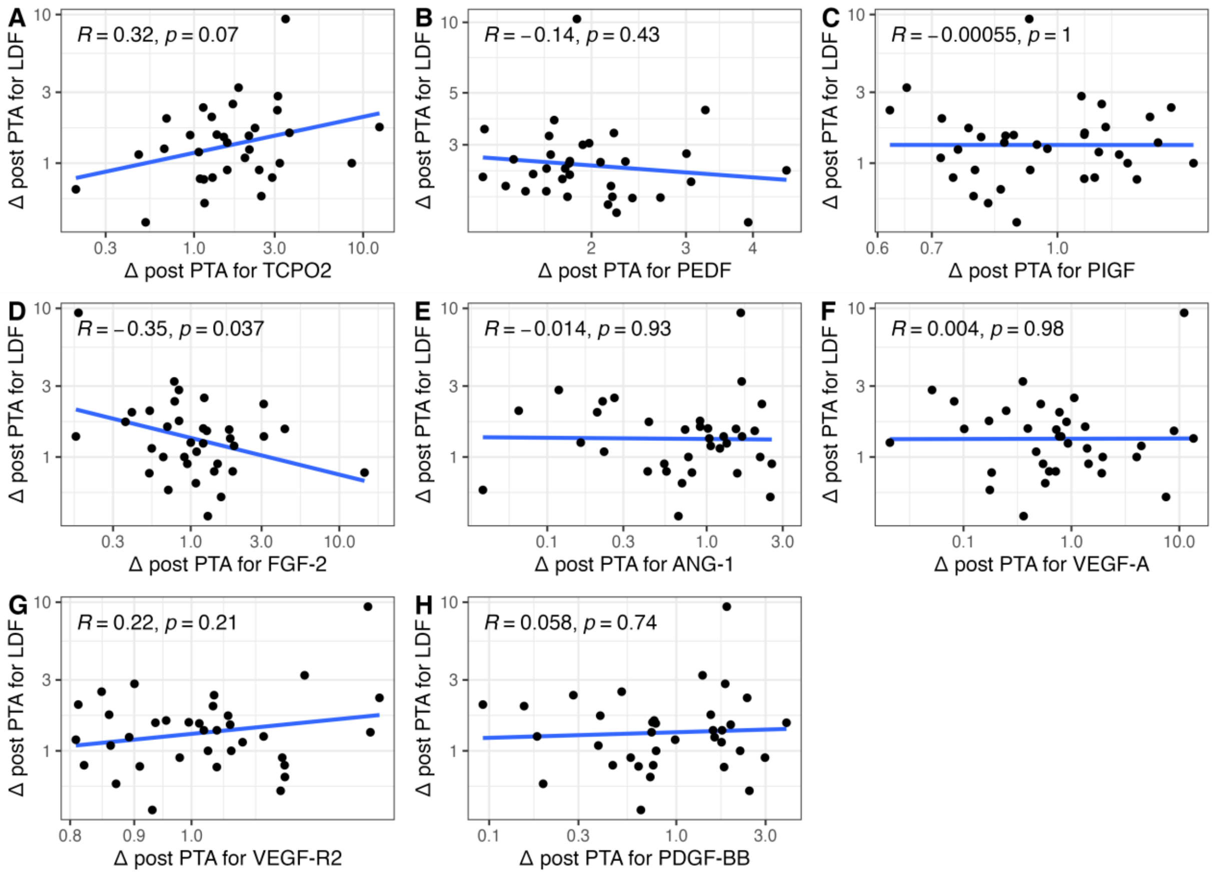

Figure S2. Scatter plots with linear fit illustrating the relationship between changes in pre- to post-intervention changes in LDF and tcpO2 (**A**) and angiogenic factor concentrations (**B** – PEDF, **C** – PlGF, **D** – FGF-2, **E** – ANG-1, **F** – VEGF-A, **G** – VEGF-R2, **H** – PDGF-BB). VEGF-A—vascular endothelial growth factor alpha/A, VEGF-R2—vascular endothelial growth factor receptor 2, FGF-2—fibroblast growth factor 2, PlGF—placental growth factor, PDGF-BB—platelet-derived growth factor-BB, PEDF—pigment epithelium-derived factor, ANG-1—angiopoietin-1, tcpO2—transcutaneous oxygen pressure, LDF—laser doppler flowmetry.
